# 3D Diatom–Designed and Selective Laser Melting (SLM) Manufactured Metallic Structures

**DOI:** 10.1038/s41598-019-56434-7

**Published:** 2019-12-24

**Authors:** Izabela Zglobicka, Agnieszka Chmielewska, Emre Topal, Kristina Kutukova, Jürgen Gluch, Peter Krüger, Cathy Kilroy, Wojciech Swieszkowski, Krzysztof J. Kurzydlowski, Ehrenfried Zschech

**Affiliations:** 1 Faculty of Mechanical Engineering, Bialystok University of Technology, 45C Wiejska Str., 15–351 Bialystok, Poland; 2 Fraunhofer Institute for Ceramic Technologies and Systems IKTS, Maria–Reiche–Strasse 2, 01109 Dresden, Germany; 3 Faculty of Materials Science and Engineering, Warsaw University of Technology, 141 Wołoska Str., 02–507 Warsaw, Poland; 4 Materialscare LTD, 10/1 Zwierzyniecka Str., 15–333 Bialystok, Poland; 5 Dresden Center for Nanoanalysis, Dresden University of Technology, 01062 Dresden, Germany; 6 National Institute of Water & Atmospheric Research Ltd., 10 Kyle Str., Riccarton, P.O. Box 8602, Christchurch, New Zealand

**Keywords:** Structural properties, Structural materials, Techniques and instrumentation, Imaging techniques, Materials science, Nanoscience and technology, Mechanical engineering

## Abstract

Diatom frustules, with their diverse three-dimensional regular silica structures and nano- to micrometer dimensions, represent perfect model systems for biomimetic fabrication of materials and devices. The structure of a frustule of the diatom *Didymosphenia geminata* was nondestructively visualized using nano X-ray computed tomography (XCT) and transferred into a CAD file for the first time. Subsequently, this CAD file was used as the input for an engineered object, which was manufactured by applying an additive manufacturing technique (3D Selective Laser Melting, SLM) and using titanium powder. The self-similarity of the natural and the engineered objects was verified using nano and micro XCT. The biomimetic approach described in this paper is a proof-of-concept for future developments in the scaling-up of manufacturing based on special properties of microorganisms.

## Introduction

Diatoms have been studied by biologists since the 18^th^ century because of their unique, intricately patterned silica cell walls (frustules) coupled with their abundance in various aquatic and terrestrial environments. Even today, frustule shape, size, patterning and structure, observed in light and electron microscopes, are still the primary characteristics to identify diatom species^1–3^. Recently, diatoms have attracted growing attention from the engineering community since their hierarchical structure and the functionality of their frustules (which is comprised of parts or two valves) have, through the long evolution of the group, achieved levels of precision not accomplished with artificial structures manufactured by humans. Natural organisms like diatoms, which are characterized by a hierarchical architecture and a large variety of morphology, have a huge potential to be utilized as original patterns for the design of advanced materials with dedicated application-specific properties. Some applications have been described in literature (see^4–6^).

The major engineering approaches based on diatoms include:

- understanding and modelling of their mechanical properties^7,8^;
- exploring the use of diatom shells in drug delivery^9,10^;
- development of a next-generation solar cells^11,12^, micro-lenses^13,14^, optical sensors and biosensors^15,16^;
- production of biofuels on a commercial scale^17^;
- formation of nanostructured metallic micro-shells using frustules as templates^18–21^.

To understand the unique properties of diatoms, their interior (hierarchical sub-structure) has been studied based on microscopic imaging of cross-sections of the shells^22,23^. Such two-dimensional (2D) visualization of biological objects is a routine task. Commonly, focused ion beam (FIB) serial cutting and subsequent scanning electron microscopy (SEM) imaging is the approach of choice for preparing and examining specimens at target sites^24^. The three-dimensional (3D) object is reconstructed from a large number of 2D images. However, the serial cutting approach has several disadvantages for the accurate representation of structures. A major drawback is the generation of preparation artefacts that result in material damage caused by the interaction of the Ga^+^ ion beam with the natural material. Particularly for elongated diatom species, the size of a frustule, which can be up to 5–6 mm, is too large for studying the whole 3D frustule structure using FIB/SEM serial cutting with reasonable effort. Furthermore, the excitation of electrons is increased at edges of a solid structure^25^, and consequently, they generate a local high brightness in the SEM image, the so–called ‘edge effect’.

In contrast to the destructive serial cutting technique, nano X–ray computed tomography (nano-XCT) provides detailed 3D information of the morphology of diatom frustules with sub-100 nm resolution^24^. Large field-of-view imaging provides an overview of the sample and allows the identification of a region of interest (ROI), and subsequent small field-of-view imaging enables the visualization of the ROI with higher resolution. Furthermore, nano-XCT at multi-keV photon energies does not require ultra-high vacuum, and therefore allows the study of diatoms at various environmental conditions.

In this paper, we describe a procedure for imaging the structure of the diatom *Didymosphenia geminata* using nano-XCT with high resolution, the generation of a CAD model and the fabrication of a self-similar, bio-inspired structure applying 3D Selective Laser Melting (SLM).

The use of several methods, including gas/solid displacement, sol-gel synthesis, polymerization and genetic/environmental manipulation, to transform biosilica into ceramics (MgO, TiO2), semi-conducting (Si-Ge) or organic scaffolds (polyaniline) structures with a retention of shape and fine features of diatom frustule structure have been reported^18,26–28^. These conversions transform the diatom frustule into a wide variety of chemistries without losing the bio-assembled 3D morphologies.

**Figure 1:**
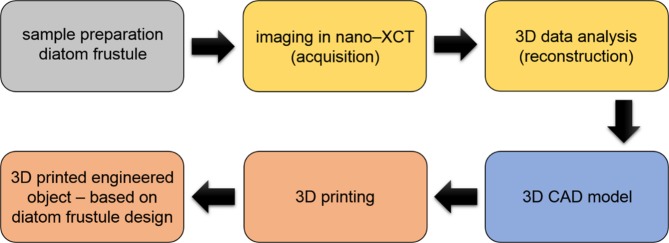
Flow diagram for 3D printing of an engineered object, based on the 3D nondestructive visualization of the natural object (diatom *Didymosphenia geminata*) using nano-XCT. The fabrication of the self-similar 3D object with bio-inspired design is based on accurate 3D data (CAD model).

In the approach presented here, we show for the first time, an entire “work-flow” (Fig. 1) from the non-destructive depiction of the interior of a diatom frustule, through the generation of a CAD model, up to the self-similar reproduction applying additive manufacturing.

## Results and Discussion

Self–similar 3D objects with bio-inspired design were fabricated using additive manufacturing based on an accurate 3D data sets (CAD model), generated based on high–resolution XCT data.

**Figure 2:**
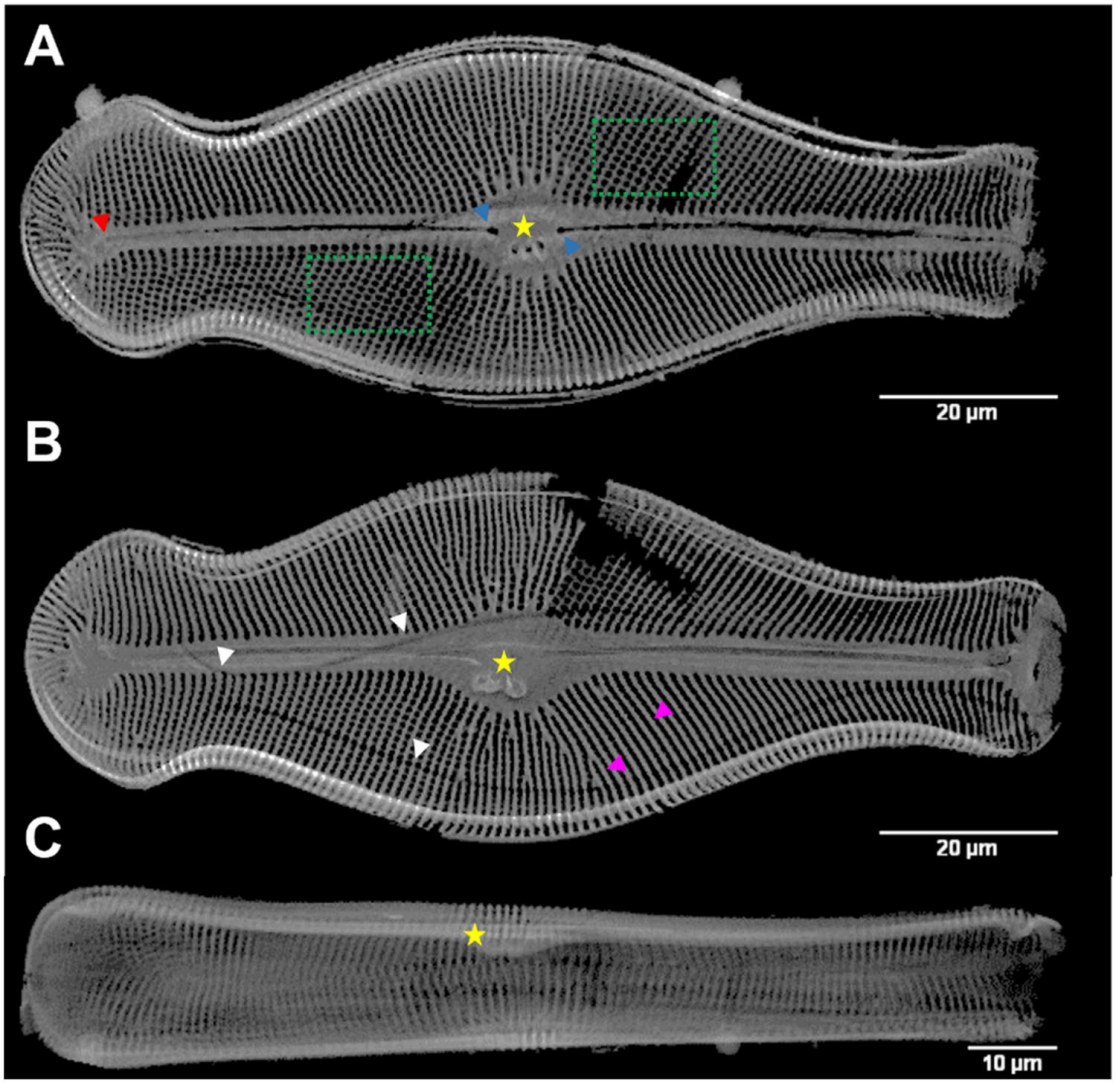
Nano-XCT of the frustule of *D. geminata* (in phase contrast imaging mode). (**A**–**C**) Slices extracted from the reconstructed 3D volume in (**A**) valve view – epitheca, (**B**) valve view of the interior – hypotheca, and (**C**) girdle view of the frustule. Yellow star: stigmata, red arrowhead: raphe distal end, blue arrowheads: raphe proximal ends, purple arrowheads: ribs, white arrowheads: girdle band, green rectangle: pores/areolae.

The structure of an entire frustule, with features such as ribs (purple arrowheads), and even areolae with sub-micrometer size (green rectangles), is clearly visible in the virtual cross-sections based on nano-XCT data (Fig. 2). Other visible characteristics of *D. geminata* included: stigmata (yellow stars), raphe with distal (red arrowhead) and proximal (blue arrowheads) ends (Fig. 2). Even a girdle band that was detached from the frustule is visible (Fig. 2, white arrowheads). These examples demonstrate that high-resolution and nondestructive visualization of morphology allows one to identify characteristic features of frustules of a particular diatom species, as well as variation within a group of representatives of the same species.

The radiograph in Fig. 2B shows a hole resulting from missing data of the two reconstructions that were carried out separately for headpole and footpole sections, needed because of the length of the frustule.

**Figure 3:**
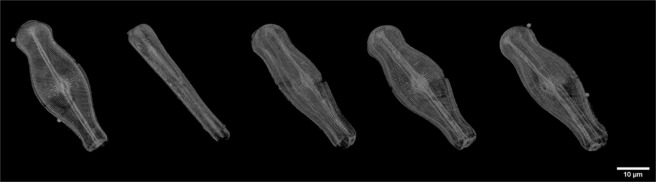
Three–dimensional (3D) visualization of frustule of *Didymosphenia geminata* based on nano-XCT imaging.

Because of the hierarchical architecture and the presence of specific internal substructures of diatom frustules, single radiographs are not sufficient for the visualization of their 3D internal structure. The 3D visualization of the obtained segmented data is presented in Fig. 3.

**Figure 4:**
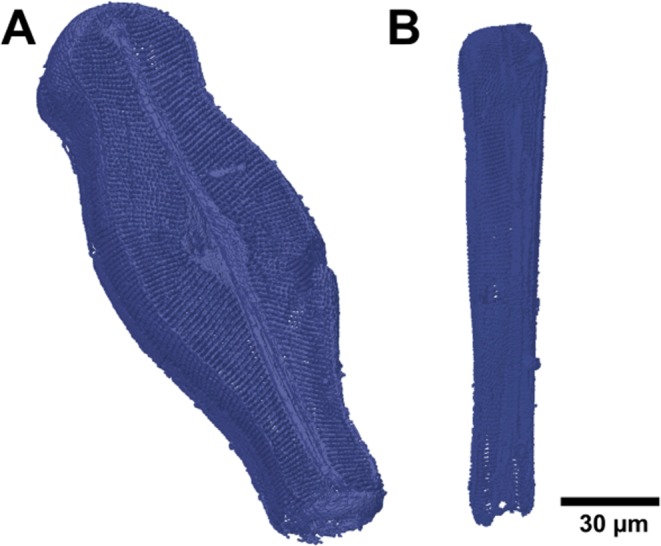
CAD model of *D. geminata* frustule prepared for 3D printing: (**A**) valve view, (**B**) girdle view.

The numerical model of the sub-structure, based on a CAD model was obtained based on the reconstructed 3D data of the complete diatom frustule structure (Fig. 4). Subsequently, the data were converted into the STL file format (*.stl, STereoLitography file), which describes the external closed surfaces of the original CAD model. The *.stl file provides the basis for the calculation of slices.

**Figure 5:**
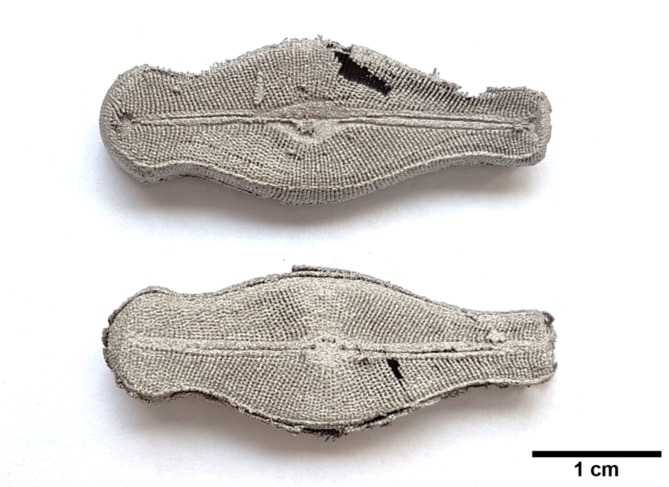
3D printed engineered object made of titanium, based on the natural diatom frustule design.

For 3D printing using the SLM process, the smallest size of a single element (detail) was fixed to 100–150 µm since the particle size of the used powder was <45 µm. Considering the selected technology and the respective equipment as well as a Ti powder with a size of 45 μm, the printed structure have larger sizes than the natural diatom frustule. In this particular case, a scaling factor of 300 was used. The 3D printed object was made of pure titanium powder, i.e. not of the natural material. Despite this change, the design of the printed “diatom frustule” remained unchanged, i.e., the printed object (Fig. 5) is self-similar to the natural object (Fig. 2).

The macroscopic observation of the 3D printed titanium object shows a hole again (upper photo in Fig. 5) – as mentioned, resulting from missing data of the two reconstructions that were carried out separately (compare to Fig. 2).

**Figure 6:**
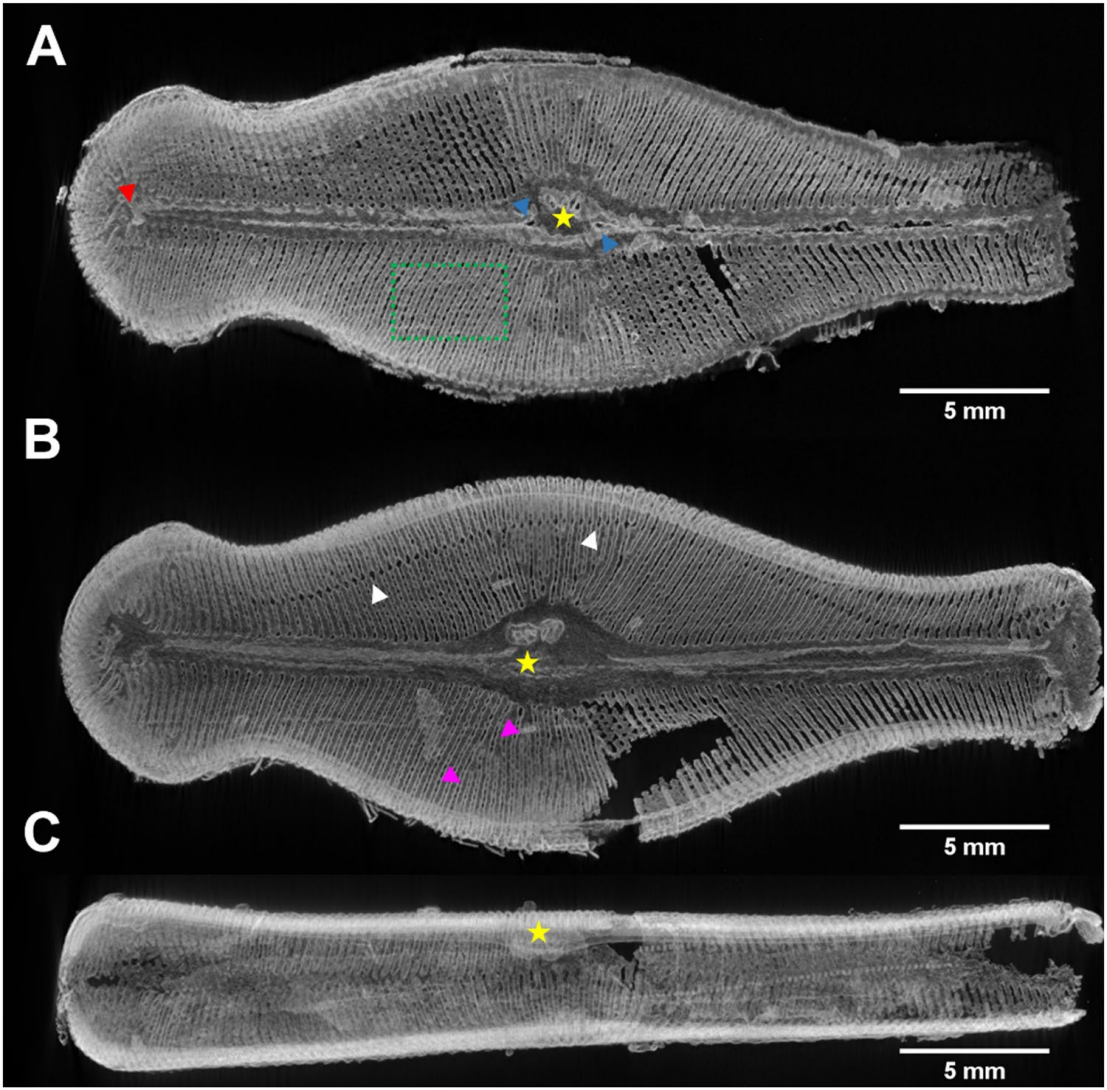
Micro-XCT results of the printed object based on the design of frustule of *D. geminata*. (**A**–**C**) Slices extracted from the reconstructed 3D volume in (**A**) valve view – epitheca, (**B**) valve view of the interior – hypotheca, and (**C**) girdle view of the “frustule”. Yellow star: stigmata, red arrowhead: raphe distal end, blue arrowheads: raphe proximal ends, purple arrowheads: ribs, white arrowheads: girdle band, green rectangle: pores/areolae.

The size of the printed object (length – ca. 3.4 cm) enables the observation of characteristic features - sub-structures of the printed object that is self-similar to the natural diatom frustule – after destructive cutting without applying microscopy (Fig. 5). However, the interior of the 3D printed self-similar object can be visualized non-destructively applying micro-XCT only (Fig. 6). Comparing the printed object (Fig. 6) with the natural diatom frustule (Fig. 2), the skeleton as well as characteristic features were preserved. Details of the diatom frustule morphology are marked in Fig. 6.

**Figure 7:**
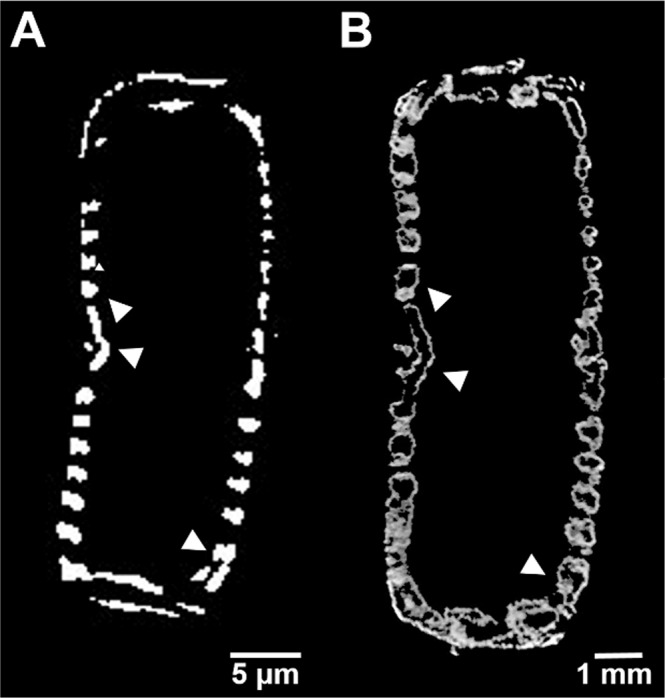
(**A**) Nano-XCT and (**B**) micro-XCT results of cross-sections of self-similar (**A**) natural and (**B**) printed objects based on the design of the frustule of *D. geminata* (arrowheads: ribs). Scaling up the frustule causes different scale bars.

Both nano-XCT of the natural object and micro-XCT of the self-similar 3D-printed object allow the visualisation of the internal structures of the objects based on virtual cross-sections. As an example, two cross-sections showing ribs are compared in Fig. 7. The discontinuity between ribs, observed on a cross-section of a natural frustule, is caused by the intervals between subsequent images.

Scanning the laser point on contour allowed us to fabricate any rib of the frustule, whereas in standard hatching some of them might be omitted (due to the absence of a laser path or occurrence between paths). For the larger (wider) ribs, the empty interior is visible. The average thickness of the wall of empty ribs was determined to be 66 ± 15 μm (n = 50), whereas the thickness of the whole ribs in the printed object was determined to be 452 ± 129 μm (n = 50).

The images obtained allow a quantitative assessment (Table 1). It was possible to imitate the natural object. Note that the engineered object is about three hundred times larger than the natural diatom frustule. The SLM technique employed for this work demonstrates a high degree of reproducibility.

**Table 1:** The mean values and standard deviations of sub-structures of *D. geminata*.

object	length	width			
		headpole	middle	footpole	single rib
diatom frustule [μm]	116 ± 2	29 ± 1	40 ± 2	20 ± 1	0.6 ± 0.1
printed object [mm]	34 ± 1	9 ± 0	12 ± 0	6 ± 0	0.2 ± 0

## Conclusions

For the biomimetic approach described in this paper, a coherent workflow for the fabrication of engineered objects that are self-similar to biological objects was developed. Nano-XCT was used to determine detailed nondestructively morphological information of the diatom frustule with high spatial resolution. This diatom frustule structure was transferred into a CAD file, which was used as input for 3D printing of the up-scaled, artificial, self-similar object which was fabricated using additive manufacturing techniques, particularly SLM. The self-similarity of the natural and the engineered objects was demonstrated using X-ray computed tomography (nano-XCT for the diatom and micro-XCT for the engineered Ti object).

The size of the biological samples such as, for example, diatom frustules (length below 150 μm) make it difficult to observe characteristic features of the specimen. Our approach of combining nano-XCT with 3D printing enhances the way of visualization of the interior biological objects such as microorganisms. The enlarged engineered object allows one to visualize the sub-structures without applying a microscope, which may broaden the knowledge in fields like biology, diatom research, as well as materials science and mechanical engineering. The potential application of the diatom-based bio-inspired nanostructures include “rib-cages” which have the potential to provide mechanical protection and to constitute an innovative reinforcement component in composite materials. Furthermore, the shape and construction of openings in diatoms (i.e. pore, areolae) could be used in gas or nutrient exchange applications.

These results provide a proof-of-concept of the workflow to engineer objects that are self-similar to natural objects. Future challenges will be to determine the mechanical properties of these frustules and to optimize the properties of macroscopic size 3D structures printed using the geometrical pattern of the bio–nano–archetype.

## Experimental Materials and Methods

### Materials

The *Didymosphenia geminata* samples were collected from the Upper Ohau River, South Island, New Zealand. Intact mats were carefully scraped from riverbed boulders, using a stiff plastic spatula, and gently rinsed in the flowing river water to release trapped sediment and other material. The fresh samples were drained for 2 h and dried at 37 °C for 48 h to prevent the growth of bacteria. Subsequently, the cells were separated from the stalks. The scraped and dried material was inserted into nylon-mesh filtration bags (Carl Roth GmbH, Karlsruhe, Germany) and immersed in distilled water for dialysis to remove dissolved salts from the natural stream waters. The dialyzed material was then sonicated for 12 h using a CD–4860 digital ultrasonic cleaner (Xiejian, Guangdong, China) in continuous mode without heating. The cells collected from the tube were boiled in 37% hydrogen peroxide (H_2_O_2_), and subsequently, in 37% hydrochloric acid (HCl) to remove organic matter and to obtain ultra–pure diatom frustules. The final suspension was washed several times with distilled water, and the clean frustules were dried in vacuum at 37 °C for 12 h.

### Experimental techniques

#### Nano X–ray computed tomography (nano–XCT) of diatom frustule

A nano-XCT tool (Xradia 100, Xradia Inc., Concord/CA, USA) operated with Cu-Kα radiation was used to image the frustule of *D. geminata*^24,29^. This tool provides the resolution needed, i.e. 50 nm, to image substructures in diatoms such as the ornamentation on the frustule, including striae (rows of pores or areolae) and ribs (i.e., interstriae) separating them. Areolae of sub-micrometer size are of particular interest. While the frustule is several tens of micrometers wide, the accumulated thickness of the silica material in the frustule was estimated to be less than 10 µm.

The rotating X-ray tube of the X-ray microscope was operated at an acceleration voltage of 40 kV with a target power of about 1200 W. Since the absorption contrast in the nano-XCT images is very low for 8 keV photons, the full-field X-ray microscopy images (radiographs) were acquired in phase contrast mode^30^. An isolated and dried frustule of *D. geminata* was mounted on the top of a needle. The complete tomographic data set comprised 801 images each, which were collected for 180°, with an exposure time of 220 s per image. Gold fiducial markers were carefully positioned on the top and in the middle part of the sample for the alignment of the individual images acquired at several tilt angles for tomographic reconstruction. The images were aligned and combined using a custom plugin in ImageJ^31^ and subsequently reconstructed using the Xradia Inc. commercial software package^30^. Due to the length of the frustule (125 µm), image acquisition and reconstruction were conducted separately for the upper (headpole, or wide end) and lower (footpole, or narrow end) parts of the frustule.

#### 3D data analysis and CAD model design

The reconstruction of a set of 2D X-ray projections (radiographs) was conducted by a filtered back projection algorithm (FBP)^32^. The reconstructed 2D image stacks - registered in ImageJ - were finally fused by stitching in order to obtain a full frustule. The final field of view was 66.5 μm with 1024 pixels, resulting in a pixel size of 65 nm. A simple threshold segmentation approach was employed to convert grey-scale images into binary images. Ultimately, the obtained 3D binary volume was transformed to a surface mesh. The quality of the surface mesh was improved using the SpaceClaim software package by closing the free edges, removing the self-intersections and making the mesh manifold in order to reduce the risk of 3D printing errors.

#### Printing of objects based on the diatom frustule design

The artificial objects based on the 3D design of the natural frustule were fabricated from spherical CP Ti (Grade 1) powder with a diameter smaller than 45 µm on a Realizer SLM50 desktop 3D printer (Realizer GmbH, Borchen, Germany). The processing was performed in an inert argon gas atmosphere, and the oxygen concentration was kept in the range of 0–0.2 vol %. The 3D model for the whole engineered object (based on diatom frustule design) consisted of 494 layers, each 25 µm thick. The objects that are self-similar to the frustule were 3D-printed based on the CAD model. The process parameters were determined such that a high manufacturing accuracy of the 3D structures, i.e. with a point distance of 20 µm, could be achieved. The exposure time was 20 µs, only the outer contour of each cross-section was melted. The low laser power of 17.5 W enabled melting of even small, irregular regions of the shell and ensured high manufacturing accuracy.

**Figure 8:**
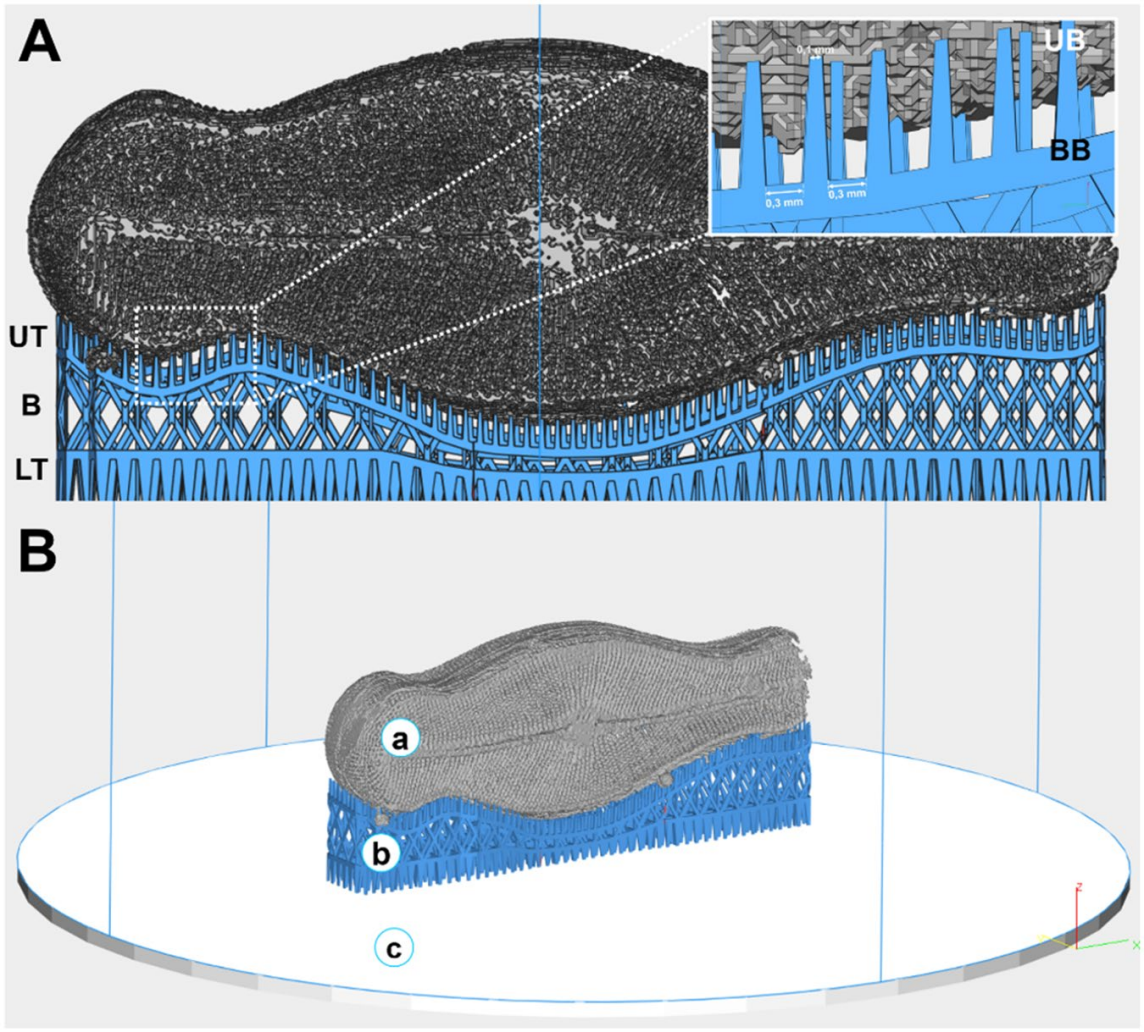
(**A**) Connection between the 3D printed engineered object and the support structure. UT – upper teeth, B – body, LT – lower teeth. Inset: magnification of the connection: UB – upper base, BB – bottom base. (**B**) Visualization of the 3D printed engineered object (a), support structure (b) and building platform (c).

The support structure was designed in a way to ensure convenient detachment of the 3D object from the platform (Fig. 8A) without machining (e.g., cutting). The truss block support structure (Fig. 8A) provided the contact to the object, yet simple detachment, using 0.1 mm wide teeth spaced 0.3 mm apart. In addition, the laser power used for the support fabrication was lower than that for the object (12.5 W, compared to 17.5 W), which results in a lower strength of the support. Such an arrangement of many thin and fine teeth placed close to each other enabled the removal of the object without damage and ensured a high-quality printed object.

#### Chemical polishing

Post-processing steps are needed to improve the surface quality and to obtain objects with high accuracy as predicted from the CAD model. The 3D objects were manually removed from the platform and the support structures (Fig. 8B) and cleaned in an ultrasonic cleaner U-507 (Zakład Urządzeń Elektronicznych “Ultron”, Dywity/Olsztyn, Poland) using distilled water (5 times for 10 minute) to remove loose powder. Subsequently, chemical polishing in a mixture of hydrofluoric acid and nitric acid (1.25 M HF/4.5 M HNO_3_) was applied for each sample using separate PTFE beakers in the ultrasonic cleaner^33^. The following chemical polishing procedure was applied:

1. Placement of the titanium object in a 20 ml PTFE beaker containing 15 ml of HF/HNO3 mixture (1 object per beaker) under sonication for 3 minutes to remove powder particles sintered to the object surfaces and to meet the targeted dimensions (from CAD model) and accuracy;
2. Removal of the acid and ultrasonic cleaning in distilled water with two solution changes, with 6 minutes sonication per water change;
3. Drying in a thermal chamber (Wamed KBC–30, Warsaw, Poland) for 2 h at 60 °C.

#### Computed tomography of a 3D printed self-similar object with bio-inspired design

Micro-XCT (customized tool at Fraunhofer IKTS, Dresden, Germany) was used to image the printed object. The micro-focus X-ray tube was operated at an acceleration voltage of 90 kV with a target power of about 3 W. The distance between source and detector was 1000 mm. The pixel size was 20 µm. The complete tomographic data set covering 360° was based on 1600 images, with an exposure time of 2 s for each individual image.
